# REDUCING THE IMPACT OF CHILDHOOD MISFORTUNE: THE ROLE OF ADULT PHYSICAL ACTIVITY ON LATER OBESITY

**DOI:** 10.1093/geroni/igz038.222

**Published:** 2019-11-08

**Authors:** Kenneth F Ferraro, Madison R Sauerteig, Monica M Williams-Farrelly

**Affiliations:** Purdue University, West Lafayette, Indiana, United States

## Abstract

This study investigates the effects of childhood misfortune and adult physical activity on later-life body mass index (BMI) and waist circumference. We use ordinary least squares regression to examine the impact of childhood misfortune (30 indicators), and adult physical activity (frequency and intensity) on waist circumference and BMI (kg/m²) using data from the Health and Retirement Study (N=5,732). Results emphasize that experiencing childhood misfortune is associated with a larger waist circumference and BMI in later life, while adjusting for social status and lifestyle variables. Adjusting for adult physical activity decreases the effect of childhood misfortune on waist circumference, suggesting mediation. The analysis reveals that the effects of childhood misfortune on BMI and abdominal adiposity are remediable. Although childhood misfortune is associated with larger waist circumference and BMI in later life, regular physical activity reduces the risk on both indicators of obesity.

